# AI‐smartphone markerless motion capturing of hip, knee, and ankle joint kinematics during countermovement jumps

**DOI:** 10.1002/ejsc.12186

**Published:** 2024-08-28

**Authors:** Philipp Barzyk, Philip Zimmermann, Manuel Stein, Daniel Keim, Markus Gruber

**Affiliations:** ^1^ Department of Sport Science Human Performance Research Centre University of Konstanz Konstanz Germany; ^2^ Subsequent GmbH Konstanz Germany; ^3^ Department of Computer and Information Science University of Konstanz Konstanz Germany

**Keywords:** AI‐technology, human movement, joint kinematics, markerless motion capture, smartphone camera

## Abstract

Recently, AI‐driven skeleton reconstruction tools that use multistage computer vision pipelines were designed to estimate 3D kinematics from 2D video sequences. In the present study, we validated a novel markerless, smartphone video‐based artificial intelligence (AI) motion capture system for hip, knee, and ankle angles during countermovement jumps (CMJs). Eleven participants performed six CMJs. We used 2D videos created by a smartphone (Apple iPhone X, 4K, 60 fps) to create 24 different keypoints, which together built a full skeleton including joints and their connections. Body parts and skeletal keypoints were localized by calculating confidence maps using a multilevel convolutional neural network that integrated both spatial and temporal features. We calculated hip, knee, and ankle angles in the sagittal plane and compared it with the angles measured by a VICON system. We calculated the correlation between both method's angular progressions, mean squared error (MSE), mean average error (MAE), and the maximum and minimum angular error and run statistical parametric mapping (SPM) analysis. Pearson correlation coefficients (r) for hip, knee, and ankle angular progressions in the sagittal plane during the entire movement were 0.96, 0.99, and 0.87, respectively. SPM group‐analysis revealed some significant differences only for ankle angular progression. MSE was below 5.7°, MAE was below 4.5°, and error for maximum amplitudes was below 3.2°. The smartphone AI motion capture system with the trained multistage computer vision pipeline was able to detect, especially hip and knee angles in the sagittal plane during CMJs with high precision from a frontal view only.

## Highlights

- Smartphone‐based movement analysis can accurately estimate joint angles during countermovement jumps.
- AI‐controlled algorithms calculate joint angles in the sagittal plane from a smartphone video in a frontal view.
- The study demonstrates the potential of AI‐controlled movement analysis with one camera for 3D kinematics.
- Future research should focus on expanding the training data to enable the analysis of more complex movements and improve temporal modeling.

## 1 INTRODUCTION

Human movement analysis is an essential aspect of various research and application fields such as medicine, sports science, rehabilitation, biomechanics, and robotics. Analyzing human movement patterns can provide insights into a range of biological and physiological processes. Movement analysis is used for injury prevention (Crowell & Davis, 2011), rehabilitation purposes (Mayr et al., 2007), as well as performance analysis (Novacheck, 1998). Traditionally, optical motion capture technologies are the gold standard for studying human movement patterns (Fukuchi et al., 2018; Liao et al., 2020; Ozkaya et al., 2018). However, these technologies are often costly, immobile, and require complex hardware and computing power, limiting their use in field studies. As a result, researchers have explored the development of low‐cost and easily accessible alternatives that can be used both inside and outside the lab.

Markerless motion capture systems show potential as tools for evaluating human motion in research and clinical settings (Armitano‐Lago et al., 2022; Mauntel et al., 2021; Moro et al., 2022; Mündermann et al., 2006; Prakash et al., 2018; Sarafianos et al., 2016). Continuing the advancement of current 3D motion capture, markerless systems could provide precise measurement of motion patterns in a practical, noninvasive manner, while also mitigating the risk of measurement‐related artifacts that are prevalent with marker‐based systems (Della Croce et al., 2005; Leardini et al., 2005). Recent progress in markerless motion capture technology has resulted in readily available and cost‐effective systems that are capable of addressing numerous queries regarding human movement.

With the availability of markerless motion capture software and systems, it is important that researchers and clinicians understand the current capabilities of these technologies and what additional considerations are required when implementing them. This includes computational and methodological approaches adopted for successful markerless motion capture. In recent years, machine learning and computer vision have witnessed significant advancements, leading to the development of markerless motion capture systems that use artificial intelligence (AI) and deep learning techniques. These systems can track body movements from video recordings without the need for specialized hardware or markers. While many systems use multiple cameras or video‐angles for their pose estimation and movement analysis (Horsak et al., 2023; Moro et al., 2020; Stenum et al., 2023; Uhlrich et al., 2022; Van Hooren et al., 2023), several systems have used single‐camera data in comparison to marker‐based motion capture systems (Aderinola et al., 2023; Lonini et al., 2022; Mercadal‐Baudart et al., 2024). The big advantage of single‐camera systems is the technical simplicity of movement analysis in a variety of environments. However, single‐camera systems in particular have shown variable results as well as several limitations in comparison to marker‐based motion capture systems. Aderinola et al. (2023) used OpenPose to calculate jump heights during countermovement jumps and reported intraclass correlations greater than 0.83. Nevertheless, they argue that current approaches to markerless motion capture are often susceptible to external factors, such as movement, lighting, and viewpoint as well as the quality of the captured videos, which can result in imprecise pose estimation and the resulting parameters. Lonini et al. (2022) investigated gait parameters (cadence, double support time, swing time, stance time, and walking speed) in stroke patients. The results demonstrated correlations of at least 0.92 for all parameters, except for swing time, which exhibited a correlation of only 0.4 and 0.66 for the nonparetic and paretic sides, respectively. Mercadal‐Baudart et al. (2024) were able to show root mean square angle errors of less than or equal to 15° estimation with the “Strided Transformer 3D pose model” for various functional movements. In this study, we have validated a novel markerless, video‐based AI motion capture system that uses deep learning techniques for motion tracking (Stein, 2020). The system (hereafter referred to as ‘Sbsq‐pose’) was developed by Subsequent GmbH, with whom this project was carried out in collaboration. The system utilizes various preexisting training datasets, including the Microsoft COCO (Lin et al., 2014), and will progressively enhance and refine its training dataset with its own collected and analyzed data. Sbsq‐pose can calculate and analyze body movements from smartphone video recordings and estimate 3D kinematics from 2D video sequences. The innovative aspect of this system is its ability to detect and analyze movements in the sagittal plane from a frontal view, which was previously impossible with traditional 2D video analysis techniques. It uses a deep learning framework based on a convolutional neural network (CNN). The CNN was trained to predict the 3D joint positions of the lower limb using 2D video frames as input. The network architecture consists of several layers, including convolutional layers that extract spatial features from the input video frames, followed by fully connected layers that map the extracted features to the 3D joint positions. Our study contributes to the development of low‐cost, accessible, and user‐friendly motion capture systems. Sbsq‐pose does not require specialized hardware or markers and is easily accessible via smartphone video recording. This accessibility and affordability will facilitate the collection of large datasets in the field, which have previously been difficult to obtain. In addition, Sbsq‐pose's ability to estimate 3D kinematics from 2D video sequences has significant implications for the analysis of motion patterns from previously recorded video footage. This capability opens up new possibilities for researchers to analyze previously recorded video data. In sports science, researchers could use the system to analyze athletic performance and develop training programs to improve athletic ability. Similarly, in rehabilitation, the system could be used to evaluate patient progress and develop rehabilitation programs tailored to individual patient needs.

One of the most commonly used movements to analyze lower extremity strength is the countermovement jump (CMJ). The CMJ is a simple movement that is often analyzed because it plays an important role in assessing neuromuscular performance in activities of daily living such as walking, climbing stairs or simply getting up from a chair or bed, or to reflect a person's overall functional status (Gruber et al., 2022). It is characterized by rapid angular displacements and large amplitude changes, particularly at the hip, knee, and ankle, mainly in the sagittal plane. The average maximum angle values relative to the joint angles of the initial posture for CMJs are approximately 90–110° for the hip, 80–100° for the knee, and 20–40° for the ankle joint (Fukashiro et al., 2005; Yoshioka et al., 2010). The CMJ is an ideal tool for validating or evaluating novel technologies due to its simplicity and ease of use. CMJ jump height has been utilized to quantify and validate smartphone applications or inertial measurement units (Balsalobre‐Fernández et al., 2015; Gruber et al., 2022; Pino‐Ortega et al., 2018) as well as markerless motion capture (Aderinola et al., 2023). This easy movement has also been instrumental in developing and evaluating markerless motion capture techniques by comparing joint positions or joint angles (Nakano et al., 2020; Needham et al., 2022) using open‐source tools such as OpenSim (Delp et al., 2007) or OpenPose (Cao et al., 2021).

In the present study, we validated Sbsq‐pose against the Vicon system by analyzing and comparing the countermovement jump (CMJ) kinematics of hip, knee, and ankle in the sagittal plane.

## 2 METHODS

### 2.1 Participants

Eleven participants (10 men and 1 woman) aged 20–42 years (*M* = 28.4, ±9.2 years) took part in the study at the Human Performance Research Center of the University of Konstanz. The participants were recruited at the University of Konstanz. The inclusion criteria were as follows: adults aged 18 years or older, regardless of sex. Willingness to sign an informed consent form and participate in the study. The exclusion criteria included were as follows: acute lower extremity injuries or an injury in the last 6 months as well as a history of lower extremity surgery within the past 12 months. The study protocol was in accordance with the Declaration of Helsinki for human experimentation and the ethical standards of the University of Konstanz.

### 2.2 Study design

**FIGURE 1:**
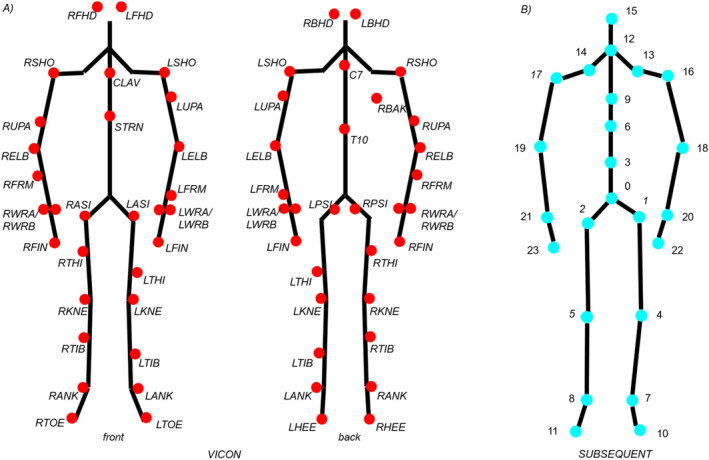
Vicon marker placement (front and back) for reflective markers (A): Right Front Head RFHD, Left Front Head LFHD, Left Back Head LBHD, Right Back Head RBHD, Clavicle CLAV, Sternum STRN, C7, Right Back RBAK, T10, Left Shoulder LSHO, Left Upper Arm LUPA, Left Elbow LELB, Left Forearm LFRM, Left Wrist LWRA/LWRB, Left Finger LFIN, Right Shoulder RSHO, Right Upper Arm RUPA, Right Elbow RELB, Right Forearm RFRM, Right Wrist RWRA/RWRB, Right Finger RFIN, Left ASIS LASI, Left PSIS LPSI, Right ASIS RASI, Right PSIS RPSI, Left thigh LTHI, Left Knee LKNE, Left Tibia LTIB, Left Ankle LANK, Left Heel LHEE, Left Toe LTOE, Right thigh RTHI, Right Knee RKNE, Right Tibia RTIB, Right Ankle RANK, Right Heel RHEE, and Right Toe RTOE; Marker model of Sbsq‐pose (B) consisting of 24 anatomical keypoints combined into a skeleton model: 0: center of pelvis, 1: center left hip joint, 2: center right hip joint, 3: lower spine, 4: center of left knee, 5: center of right knee, 6: central spine, 7: center of left ankle, 8: center of right ankle, 9: upper spine, 10: center point of left toes, 11: center point of right toes, 12: neck, 13: center point of left clavicle, 14: center point of right clavicle, 15: head center, 16: center of left shoulder joint, 17: center of right shoulder joint, 18: center of left elbow joint, 19: center of right elbow joint, 20: center of left wrist, 21: center of right wrist, 22: center of left palm, and 23: center of right palm.

Anthropometric measurements including height, weight, leg length, knee width, ankle width, elbow width, and hand thickness were obtained from each participant at the beginning of the session. Participants then completed a standardized warm‐up protocol consisting of 30 s of jumping jacks, three submaximal CMJs, three maximal CMJs, and 30 s of high knee running. Exercises were performed in a row with a 30 s break in between each exercise. Following, all participants performed eight countermovement jumps. They were instructed to stand upright with their weight evenly distributed over both feet and place hands on their hips. After ensuring the proper position, the participants were instructed to squat down until the knees were bent at approx. 90° and then without a break immediately jump as high as possible. In the flight phase, they were asked to keep their legs straight and land on both feet simultaneously (McMahon et al., 2018). Jump data were recorded simultaneously using a smartphone (Apple iPhone X with 4K resolution and a capture rate of 60 frames per second) mounted on a tripod and placed in front of the participants as well as with 12 Vicon T40‐S cameras. The Vicon motion capture system is considered the gold standard for optical motion tracking due to its high accuracy (Ozkaya et al., 2018). Forty‐three reflective markers (14 mm) were placed on anatomical landmarks (Figure 1A) according to the Plug‐in Gait (PiG) model (Vicon Motion Systems, Oxford Metrics Group Ltd). Similarly, the AI‐software extracted 24 anatomical keypoints skeletal structures (Figure 1B) from the smartphone videos to estimate joint kinematics. To synchronize the systems, the maximum hip and knee flexion and extension during the jump movement is determined for both the Vicon and markerless methods. We use a least squares fit to determine the temporal offset between the two systems based on these positions.

### 2.3 Material and data acquisition

#### 2.3.1 Vicon motion capture

The 3D motion capture system from Vicon (Vicon MX, Oxford Metrics, Oxford, UK) consisted of 12 Vicon‐T40S infrared cameras with a sampling rate of 100 Hz and the software Vicon Nexus (Version 2.12, Oxford Metrics, Oxford, UK). After initial calibration of the system, small reflectors with a diameter of 14 mm were used. The kinematics of the segments and joints were calculated using the PiG model, which is employed in Vicon Nexus. This model utilizes marker data to build a skeletal model and define segments (e.g., thigh, shin, etc.). The model determines the relative orientation of each joint center and calculates the resulting joint angles, utilizing anthropometric data, marker positions, and the defined segments (Figure 1A).

### 2.4 Subsequent machine learning model

The AI‐based skeleton reconstruction tool provided by Subsequent GmbH has been built upon a multistage computer vision pipeline consisting of multiple machine‐learning models. This pipeline took a single video recording as an input and produced a time series of skeleton models for each person in the scene. In contrast to previous methods (Cao et al., 2021), the approach of Subsequent not only estimated two‐dimensional skeleton data in the coordinate space of the input image but also estimated the absolute‐scale three‐dimensional pose information. The overall process operated in two major phases. First, a single‐shot person detection model based on a custom CNN was used to detect the person in the input video and estimated bounding boxes for all visible people in each individual video frame. The training dataset comprised a diverse range of publicly available datasets that included the MSCOCO (for everyday movements and interactions), AIST++ (for dance movements and performances), as well as custom in‐house datasets (for sports movements, such as running or kicking a ball, particularly in team sports), which collectively total over 10 million images. Using this bounding box information, a cropped section of the video frame was extracted and rescaled to a standardized resolution. Afterward, a custom‐developed multistage CNN was used to predict the camera‐relative 2D‐ and 3D‐locations of 24 statically defined skeleton keypoint locations for each cropped person. To enable a comparison with the Vicon data, Sbsq‐pose initially identifies the consistent keypoints between the two skeletal models. Subsequently, the missing keypoints for each skeleton are determined by weighted interpolation of the position of the surrounding keypoints belonging to the same bones. For example, the neck keypoint is calculated from the shoulder and clavicle keypoints and the pelvic center keypoint is calculated from the RASI, LASI, RPSI, and LPSI keypoints. The reconstruction of 3D location information from a single 2D video frame necessarily involved geometric ambiguities resulting from the projection on the video plane. Therefore, the model integrated visual features at multiple resolution scales as well as implicitly learned statistical information about plausible body poses and kinematic restrictions of real‐world human movements in order to reconstruct the most likely skeleton representation that matches the given input image. This set of statistical priors resulted in a model that is robust against perspective changes as well as partial occlusions and changes in visual appearance such as different types of clothing, lighting variations, or video compression artifacts. The resulting keypoint information was estimated in normalized camera‐relative coordinates, and additional information about the camera properties, such as focal length and sensor size, was used to obtain metric‐scale three‐dimensional positions for each skeleton keypoint. Finally, joint angles are calculated by selecting the two neighboring keypoints in the respective kinematic chain for each joint keypoint. For example, the hip and ankle keypoints are used for the knee joint angle. The angles are then calculated from the dot product of the vectors between the current keypoint and its two neighboring keypoints.

### 2.5 Statistical analysis

To validate the accuracy of Sbsq‐pose (Build‐number 8358, trained on 2022‐09‐09), we compared the angular response for hip flexion/extension, knee flexion/extension, as well as dorsi‐ and plantarflexion over the entire movement to that of the Vicon motion capture system. We conducted all statistical analyses using Python scripts and reported the mean and standard deviation (±SD). To compare the angular progressions of both systems, we calculated Pearson correlation coefficients (r), the mean square error (MSE), and the mean average error (MAE). We compared each data point recorded bySbsq‐pose with the corresponding data point of the Vicon analysis, resulting in average correlations and errors. We calculated both MSE and MAE, as MSE provides more information on the overall error and is more sensitive to outliers, whereas MAE provides a measure of the average magnitude of the differences between both systems, making it more robust to outliers (Rashidi et al., 2023). To assess the difference between the systems over each individual kinematic time series, we used statistical parametric mapping (SPM) (Morais et al., 2023). Furthermore, the errors for the minimum and maximum amplitudes of angular responses were calculated.

## 3 RESULTS

**FIGURE 2:**
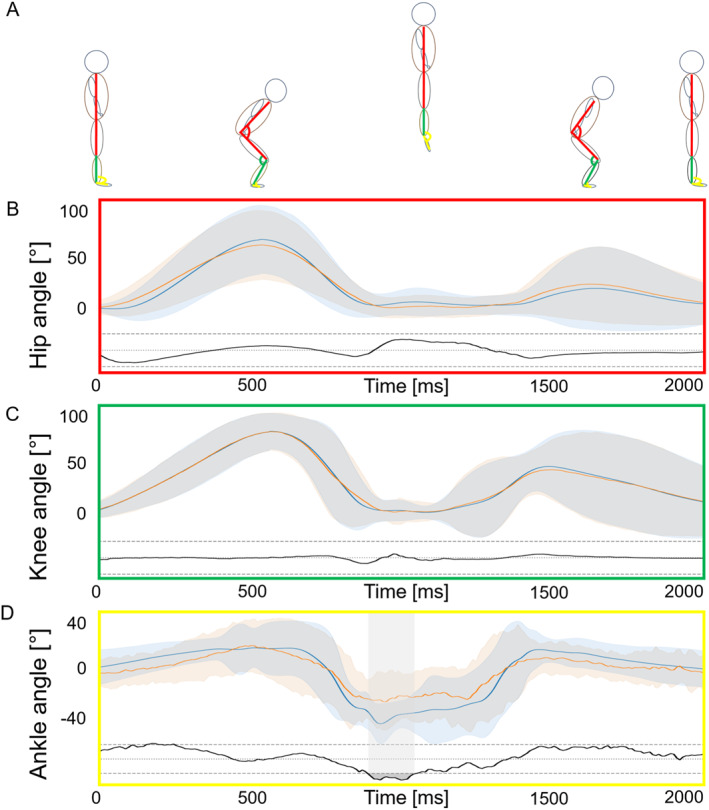
(A) Schematic depiction of the countermovement jump movement and the measured angles; mean, (B) hip, (C) knee, and (D) ankle angle in the sagittal plane of all subjects during CMJ. Zero degrees indicate full hip and knee extension and neutral ankle position. *Y*‐axis shows time in ms and *X*‐axis shows the angle in degrees. Results for Vicon Nexus are shown in blue, whereas results for the AI smartphone motion capture are displayed in orange. Results for statistical parametric mapping are shown at the bottom of each graph (gray bar indicates significance) as the corresponding *t*‐test statistics in relation to the critical threshold (black dashed lines and *p* = 0.05).

Table 1 Presents the results of the comparison between Sbsq‐pose and the vicon system. The Pearson correlation coefficients (r) for hip flexion/extension, knee flexion/extension, and dorsi‐ and plantarflexion during the entire movement were all greater than or equal to 0.87. The maximum MSE and MAE were 5.7° (±1.3) and 4.5° (±1.1) for the ankle, respectively. The maximum and minimum measured angles of both systems differed by less than 3.2°. The group analysis conducted using SPM to determine significant differences between the time‐continuous measurements of the two systems, using a *t*‐test metric, only revealed significant differences for the ankle angular progression (Figure 2). SPM results and angular progressions at the individual subject level are shown in the Appendix.

**TABLE 1:** Statistical results for the group (*n* = 11), with the average correlation (Corr.) of the respective angles of both measurement methods (Pearson's r, mean squared error (MSE), MAE, and the absolute error in the minimal (Angle_min__error) and maximal (Angle_max__error) joint angles).

	Hip	Knee	Ankle
Correlation [r]	0.96 ± 0.04	0.99 ± 0.01	0.87 ± 0.08
MSE [°]	3.2 ± 0.8	2.7 ± 1.2	5.7 ± 1.3
MAE [°]	2.6 ± 0.6	2.1 ± 0.9	4.5 ± 1.1
Angle_min__error [°]	2.7 ± 1.3	1.9 ± 1.1	3.2 ± 2.5
Angle_max__error [°]	1.5 ± 0.9	1.0 ± 0.6	2.9 ± 1.9

## 4 DISCUSSION

This study compared kinematics during CMJs measured by a novel markerless smartphone‐based motion capture system with those captured by the gold standard marker‐based Vicon motion capture system. The results showed that Sbsq‐pose achieved both high validity, as indicated by Pearson's correlation coefficient, and high accuracy, as indicated by MSE and MAE between both systems (Table 1). The SPM analysis of the entire group only revealed significant differences between the systems in the ankle analysis (Figure 2). There were greater differences at a single‐subject level. We also calculated the absolute error in the maximum and minimum angle amplitude throughout the movement. The segment angles showed similarity in 3D body segment pose estimates, with MSE values of less than 6° for all angles (3.2°, 2.7°, and 5.7° for hip, knee, and ankle, respectively) and MAE values of less than 5° for all angles (2.6°, 2.1°, and 4.5° for hip, knee, and ankle, respectively). Compared to previously reported differences between markerless and marker‐based systems, the measured values were comparable. Needham et al. (2022) reported mean differences of ≤3° for hip, knee, and ankle angles when comparing jump kinematics using an OpenSim‐based markerless model with a marker‐based system (Oqus, Qualisys AB, Gothenburg, Sweden). However, they did not perform an SPM analysis or otherwise examine where statistical differences occurred in specific moments of the movement sequence. Both Horsak et al. (2023) and Van Hooren et al. (2023) reported angular differences of ≥5.0° for hip, knee, and ankle angles during walking or running when comparing either the open‐source OpenCap system (Horsak et al., 2023) or a custom‐trained (DeepLabCut) and an existing (OpenPose) model (Van Hooren et al., 2023) with a Vicon system. Horsak et al. (2023) discussed the possibility that additional cameras, particularly in the sagittal plane, could improve accuracy, although some studies failed to demonstrate this to date (Moro et al., 2020; Stenum et al., 2023; Uhlrich et al., 2022). Interestingly, the studies using video‐based approaches often use at least two cameras, usually in a sagittal or an oblique frontal‐sagittal view, to capture their data, whereas only one camera was used in this study in a frontal view and still produced similar or even better results. Comparisons with previous single‐camera approaches are mostly difficult as either other movements or other statistical means were used to compare the markerless system with the marker‐based system. Aderinola et al. (2023) only used intraclass correlation and Bland–Altman analysis to compare the jump height calculated by both systems. They did not evaluate pose estimation or angle progression during the movement, which makes it challenging to validate the accuracy of the single‐camera approach or to transfer the results to other movements. Lonini et al. (2022) were unable to obtain or calculate 3D models from their 2D video sequences, which prevented the analysis of spatial gait parameters. The only study that included CMJ kinematics was conducted by Mercadal‐Baudart et al. (2024). In this study, the comparable angles showed RMSEs of less than or equal to 5° for the knee and ankle and ≤6° for the hip but the authors noted that multiple‐camera systems have so far been able to show better results. In contrast, our results demonstrated superior performance for hip and knee angles (3.2° and 2.7°, respectively), while our ankle angle results exhibited greater variability (5.7°).

Since the underlying deep‐learning model implicitly learns which postures are more likely to occur and which are not based on previously entered training data, errors in pose estimation can occur for movements that are less familiar to the system than others. The training data mainly consists of slow movements, which combined with the system's attempts to estimate keypoints and resulting sagittal angles from frontal video recordings can lead to a reduction in the amplitude of angle curves, especially for fast movements. However, an average absolute error of less than 3.2° for both minimum and maximum amplitude highlights the accuracy and consistency of the novel motion capture system in capturing joint angles during a CMJ.

Upon examining the average maximum angle values, it is evident that the knee and ankle angles (approximately 85° and 25°, respectively) align with the literature (Fukashiro et al., 2005; Yoshioka et al., 2010). However, the hip angle (approximately 60°) deviates significantly from the average values (approximately 90–110°). It is important to note that while Fukashiro et al. (2005) conducted a study on male intermediate‐level Australian football players and Yoshioka et al. (2010) used a computer simulation, we did not assess the athletic level initially nor consider the movement execution of the CMJ. This may have led to incorrect execution of movements, especially in more extensive hip flexion. The average angle progression of the Vicon data appears to support this.

Figure 2 illustrates significant differences between the two systems at the group level only for the ankle angle during take‐off and the first phase of flight. However, the SPM analysis of the individual participants (see appendix for the corresponding figures Figure A1) revealed more significant differences. Particularly during take‐off and the initial flight phase, there were significant differences in hip and knee angles for some participants. The greatest differences were observed in the ankle joint. Four participants showed significant deviations during take‐off, seven participants during the flight phase, and one participant each during landing or after the end of the movement. As can be seen in Figure 2D (ankle joint angle progression), certain analyses result in greater inaccuracies than others. One possible explanation for why certain analyses lead to greater inaccuracies than others is that the model estimates the position of each joint with a certain degree of uncertainty. Depending on the length of the segments used, the position error has a greater or lesser effect on the angular error. For the ankle joint, the distance from ankle to ball of foot is relatively short, so the deviation is greater. These higher inaccuracies for foot estimation are in line with findings from other studies (Horsak et al., 2023); however, this error will steadily decrease with further training data input and improvement of the analysis and will align itself proportionally with the results of the other angle progressions.

The study has several limitations for both the marker‐based and markerless motion capture systems, which represent different types of errors that could affect the estimation of joint angles. First, the study sample is comparatively small and statistical significance and should, therefore, be considered with caution. On the other hand, we were able to show excellent results for the angle trajectories in all specified body regions and are, therefore, confident that the system can also be used in in‐field studies for larger‐scale studies. However, it should be emphasized that the motion we studied is rather simple, since it is only flexion and extension of certain body regions. Although the tool used in this study for pose estimation can calculate data in other planes, sagittal data were explicitly chosen to demonstrate that angles in regions not visible to the camera can also be calculated. Furthermore, as vertical jumps require a longitudinal shift of the body's center of gravity, the sagittal angles of the lower extremities are the decisive parameters for performing a CMJ (King & Cipriani, 2010). In the further course of our research, we will investigate rotational movements and much more complex movements in general to show the comparison of the new AI‐tool with the current gold standards. In measurements and analysis already carried out on golf swings and complex routines on the parallel bars and somersault movements on the floor, we were able to show good results for the most part, but the motion detection is still significantly less accurate for less familiar motion components or for those that have not been “fed” into the system beforehand than for motion components that are “known” to the system. Upcoming analysis aspects will include the integration of additional training data, derived features, such as velocities/accelerations, and improved temporal modeling.

## 5 CONCLUSION

Overall, the high correlation coefficients and low MAE and MSE values demonstrate that the Subsequent system is able to provide valid joint angle data, here especially for hip and knee angles in the sagittal plane during CMJs and therefore can be a viable alternative to traditional motion capture systems. Nevertheless, it still has to be determined whether the system can also measure 3D movement patterns, such as rotational movements, and if it can detect accurately enough angles in the frontal or transversal plane such as varus or valgus knee alignment from a camera positioned in the sagittal plane. This study should be considered as a first step to show the potential of this new tool as a valuable asset for researchers and clinicians who require valid and reliable motion data but may not have access to expensive traditional motion capture systems.

## CONFLICT OF INTEREST STATEMENT

The authors declare that they have no conflicts of interest.
